# Knowledge, Attitudes, and Practices Toward Diabetic Retinopathy in Pregnant Women With Diabetes Among Antenatal Care Physicians in Al-Ahsa, Saudi Arabia: A Cross-Sectional Study

**DOI:** 10.7759/cureus.91595

**Published:** 2025-09-04

**Authors:** Zainab Al Jaziri, Zahraa K Alshayeb, Aisha A Bubshait, Mostafa Alhodibi

**Affiliations:** 1 Family Medicine, Al-Ahsa Health Cluster, Al-Ahsa, SAU; 2 Family Medicine, Al-Ahsa Health Cluster, Alfudhool Primary Health Care Center, Al-Ahsa, SAU

**Keywords:** antenatal care, attitude, diabetic retinopathy, knowledge, practice, pregnancy, saudi arabia, screening

## Abstract

Background

Diabetic retinopathy (DR) is a major complication of diabetes mellitus, and its progression may accelerate during pregnancy. Antenatal care (ANC) physicians play a critical role in the screening and management of DR in pregnant women. However, limited evidence exists regarding their knowledge, attitudes, and practices (KAP), particularly in regions such as Al-Ahsa, Saudi Arabia.

Objectives

The objective of this study is to assess the KAP of ANC physicians in Al-Ahsa regarding DR during pregnancy and to explore associations with demographic and professional characteristics.

Methods

A cross-sectional study was conducted among ANC physicians (N = 121) in Al-Ahsa from June 2024 to December 2024. Data were collected using a validated, self-administered questionnaire developed by the researchers. The questionnaire consisted of four sections: demographics, knowledge (multiple-choice/true-false items), attitudes (Likert scale), and practices (self-reported behaviors).

Results

Among the 121 participants, the mean age was 34.3 ± 7.4 years, and 92 (76.2%) were female. Overall knowledge of DR during pregnancy was moderate, attitudes were neutral, and practices were suboptimal. Notable gaps were observed in knowledge of appropriate DR screening intervals, along with misconceptions regarding BMI, maternal age, and the use of oral hypoglycemic agents as primary risk factors. Only one-third of participants consistently engaged in patient education or referral practices. Physicians with more experience and specialized training demonstrated greater awareness and better adherence to recommended DR screening protocols.

Conclusions

ANC physicians in Al-Ahsa exhibited moderate knowledge, neutral attitudes, and inadequate practices regarding DR during pregnancy. Targeted educational initiatives and system-level interventions are essential to improve adherence to DR screening guidelines and reduce the risk of pregnancy-related vision loss.

## Introduction

Diabetes mellitus (DM) represents a growing global health crisis, with prevalence rapidly increasing worldwide. According to the International Diabetes Federation, diabetes currently affects 537 million people globally, including 73 million in the Middle East and North Africa region, a figure projected to rise to 135.7 million by 2024. In Saudi Arabia, DM affected 17.7% of the total population in 2021 [1]. Diabetes can lead to a range of complications that vary in severity and impact multiple organ systems. Among these, diabetic retinopathy (DR) is a major neurovascular complication and the leading cause of blindness among adults in developed countries. DR is classified into two main forms: nonproliferative DR (NPDR) and proliferative DR (PDR), distinguished by the absence or presence of neovascularization, respectively [2].

Pregnancy introduces physiological changes that can significantly influence the course of DR in women with preexisting type 1 or type 2 diabetes [3,4]. Hormonal, metabolic, and hemodynamic shifts during gestation may accelerate the development or progression of DR [5]. Estimates indicate that DR is already present at the time of the initial antenatal ophthalmic examination in 57-62% of women with type 1 diabetes and 17-28% of those with type 2 diabetes [6]. Approximately 6% of these women may progress from NPDR to PDR during pregnancy [7]. Although some DR changes may regress postpartum, the risk of irreversible vision loss underscores the importance of vigilant screening and management during pregnancy and the postpartum period [8]. Key risk factors for DR progression in pregnancy include longer duration of diabetes, advanced baseline retinopathy, poor glycemic control, rapid improvement in glucose levels, hypertension (including preeclampsia), and diabetic nephropathy [9-11].

Antenatal care (ANC) physicians, including general practitioners, family medicine specialists, and obstetricians, are on the frontline of maternal care and play a pivotal role in identifying and referring pregnant women with diabetes for ophthalmologic evaluation [12,13]. Effective management requires timely DR screening, patient education, optimization of glycemic and blood pressure control, and close coordination with ophthalmologists [3,12]. International guidelines, including those from the American Academy of Ophthalmology (AAO), the American Diabetes Association (ADA), and the UK’s National Institute for Health and Care Excellence (NICE), recommend that women with preexisting diabetes undergo a dilated eye examination before conception or during the first trimester, followed by periodic assessments throughout pregnancy based on disease severity [6,14].

This study aims to assess the knowledge, attitudes, and practices (KAP) of ANC physicians in Al-Ahsa regarding DR during pregnancy.

## Materials and methods

Study design and setting

We conducted a quantitative, descriptive, cross-sectional, questionnaire-based study to assess the KAP related to DR during pregnancy among ANC physicians in Al-Ahsa, Eastern Province, Saudi Arabia. Data collection was carried out between June 2024 and December 2024 across primary health care centers and hospitals providing ANC services, including Maternity and Child Hospital, King Faisal Hospital, Al-Omran General Hospital, and Al-Jafer Hospital.

Study population and sampling

Eligible participants included physicians currently providing ANC, general practitioners, family medicine physicians, and obstetrics/gynecology consultants, specialists, or residents. The total target population was 150. A convenience sampling method was used to include 121 participants, with voluntary participation obtained after clarifying the study’s purpose and securing online informed consent. Physicians not involved in ANC, unavailable during the study period, or unwilling to participate were excluded.

Data collection

Data were collected through an online questionnaire created by the authors and validated by three gynecologists and three family medicine consultants. The questionnaire was prepared using Google Forms and distributed electronically to participants during morning meetings at their workplaces. A pilot study was conducted among 30 physicians (20% of the target population), who were excluded from the final analysis. The pilot study confirmed the reliability of all sections, with Cronbach’s alpha values exceeding 0.70 across all KAP domains.

The questionnaire included four sections. The first collected demographic and personal information, including age, gender, education level, workplace, years of experience, marital status, and number of children. The second assessed knowledge regarding the pathophysiology of DR, risk factors, screening intervals, and treatment options. The third examined attitudes toward DR, including the importance of routine ophthalmic examinations, the role of ANC physicians in DR management, and adherence to workplace protocols. The fourth addressed practices, including patient education about DR during pregnancy, assessment of visual complaints, referral to an ophthalmologist, follow-up on ophthalmologist reports, and compliance with specific guidelines for managing DR in pregnancy (Appendix A, Appendix B, Appendix C, Appendix D).

Scoring and data analysis

Data were analyzed using IBM SPSS Statistics for Windows, Version 28.0 (Released 2021; IBM Corp., Armonk, NY, USA). Continuous variables were presented as means with standard deviations, while categorical variables were presented as frequencies and percentages. Normality was assessed before further analysis. Independent t-tests were used to examine relationships between KAP domain scores and categorical variables with two levels. For categorical variables with more than two levels, ANOVA was employed. A p-value of ≤0.05 was considered statistically significant. KAP domain scores were categorized as low (<60%), moderate (60-79%), or good (≥80%) based on Bloom’s cutoff points. Descriptive statistics summarized categorical variables (frequencies and percentages) and continuous variables (means ± SD).

Ethical considerations

The study was approved by the Institutional Review Board of Maternity and Child Hospital, Al-Ahsa (IRB #MCH-H-05-HS-137). Participation was voluntary, and all responses were anonymous and confidential.

## Results

Table 1 shows that the study included 121 participants, the majority of whom were female (92; 76%), with a mean age of 34.24 years. Most participants were married (97; 80.2%) and had children (97; 76.9%). More than three-quarters (94; 77.7%) were Saudi nationals, and the majority worked in hospitals (105; 86.8%). Regarding professional background, a considerable proportion were board residents (38; 31.4%) or board-certified in family medicine (59; 48.8%). In terms of experience, 51 participants (42.1%) had 2-5 years of general experience, and 47 (38.8%) had 2-5 years of ANC experience.

**Table 1:** Sociodemographic characteristics of participants (n = 121)

Sociodemographic characteristic	Category	Frequency	Percentage (%)
Sex	Male	29	24
	Female	92	76
Age (mean ± SD)	34.24 ± 7.41	-	-
Age group	<30 years	32	26.4
	30-50 years	82	67.8
	>50 years	7	5.8
Nationality	Saudi	94	77.7
	Non-Saudi	27	22.3
Specialty	General practitioner	30	24.8
	Board resident	38	31.4
	Specialist	30	24.8
	Consultant	23	19
Degree	MBBS	45	37.2
	Diploma	8	6.6
	Family medicine board-certified	59	48.8
	Obstetrics and gynecology board-certified	9	7.4
Years of experience in general practice	<1 year	42	34.7
	2-5 years	51	42.1
	6-10 years	28	23.1
Years of experience in ANC assessment	Less than 1 year	45	37.2
	2-5 years	47	38.8
	6-10 years	29	24
Workplace	Primary health care center	16	13.2
	Hospital	105	86.8
Marital status	Single	21	17.4
	Married	97	80.2
	Divorced/widowed	3	2.5
Children	Yes	93	76.9
	No	7	5.8
	Not applicable (single)	21	17.4

Table 2 shows that most participants, 89 (73.6%), correctly recognized that pregnancy is not protective against DR, and 76 (62.8%) identified pregnancy as a risk factor for the development or progression of the condition. About 70% of participants believed that major hormonal changes during pregnancy, such as elevated levels of human placental lactogen, estrogen, and progesterone, play a significant role in worsening DR. Additionally, 45 (37.2%) acknowledged that vascular changes induced by these hormones also contribute to disease progression.

**Table 2:** Knowledge items ^*^ True answer DM, diabetes mellitus; DR, diabetic retinopathy; PDR, proliferative diabetic retinopathy; VEGF, vascular endothelial growth factor

Items		Yes	No	I don’t know
Pregnancy is considered protective against DR.	N	3	89	29
	%	2.5	73.6^*^	24
Pregnancy is a risk factor for DR.	N	76	21	24
	%	62.8	17.4^*^	19.8
Theories behind the progression of DR in pregnancy				
Hormonal changes include increased plasma levels of human placental lactogen, estrogen, and progesterone hormones.	N	85	26	10
	%	70.2^*^	21.5	8.3
Vascular changes are induced by the elevated levels of estrogen, progesterone, and human placental lactogen.	N	45	61	15
	%	37.2^*^	50.4	12.4
Is type 1 diabetes the most prone to developing DR?	N	11	89	21
	%	9.1^*^	73.6	17.4
Is type 2 diabetes the most prone to developing DR?	N	29	73	19
	%	24	60.3^*^	15.7
Is gestational diabetes the most prone to developing DR?	N	38	54	29
	%	31.4	44.6^*^	24
Which one contributes as a risk factor to the worse progression of DR during pregnancy?				
Long duration of DM	N	96	18	7
	%	79.3^*^	14.9	5.8
History of retinopathy before conception	N	70	23	28
	%	57.9^*^	19	23.1
Poor glycemic control	N	86	11	24
	%	71.1^*^	9.1	19.8
Hypertension, either preexisting or pregnancy-related, and preeclampsia	N	114	3	4
	%	94.2^*^	2.5	3.3
BMI more than 25	N	111	4	6
	%	91.7	3.3^*^	5
Multiparity	N	112	6	3
	%	92.6	5.0^*^	2.5
Older age	N	95	14	12
	%	78.5	11.6^*^	9.9
Using an oral hypoglycemic agent	N	78	26	17
	%	64.5	21.5^*^	14
Answer regarding the frequency of ophthalmic examination in diabetic pregnant ladies				
All pregnant ladies with preexisting diabetes should be screened for DR once during pregnancy.	N	49	46	26
	%	40.5	38.0^*^	21.5
All pregnant ladies with preexisting diabetes should be screened for DR each trimester.	N	96	14	11
	%	79.3	11.6^*^	9.1
All pregnant ladies with preexisting diabetes should be screened for DR once before conceiving, in early pregnancy, and in each trimester, then after accordingly.	N	30	62	29
	%	24.8	51.2^*^	24
Is a history of visual disturbance the best method to identify DR in pregnant women with diabetes?	N	56	57	8
	%	46.3	47.1^*^	6.6
Quick visual acuity and visual field assessment is the best method to screen pregnant women with diabetes regarding DR.	N	41	63	17
	%	33.9	52.1^*^	14
Detailed retinal examination with an ophthalmologist is the best method to screen pregnant women with diabetes regarding DR.	N	74	24	23
	%	61.2^*^	19.8	19
Treatment options for a pregnant lady with PDR during pregnancy				
Observational	N	47	39	35
	%	38.8	32.2^*^	28.9
Wearing glasses	N	28	50	43
	%	23.1	41.3^*^	35.5
Panretinal laser photocoagulation	N	35	37	49
	%	28.9^*^	30.6	40.5
Focal laser	N	23	39	59
	%	19	32.2^*^	48.8
Intravitreal steroids	N	18	47	56
	%	14.9	38.8^*^	46.3
Intravitreal anti-VEGF	N	35	29	57
	%	28.9	24.0^*^	47.1

Regarding diabetes type, only 11 (9.1%) correctly identified type 1 diabetes as the most prone to DR, while 73 (60.3%) disagreed with the misconception that type 2 diabetes carries the greatest risk. Similarly, 54 (44.6%) understood that gestational diabetes is not the most significant risk factor. Participants recognized several factors contributing to the worsening of DR during pregnancy, including long duration of DM (96, 79.3%), a preconception history of retinopathy (70, 57.9%), poor glycemic control (86, 71.1%), and the presence of hypertension or preeclampsia (114, 94.2%). In contrast, only a small proportion mistakenly considered a BMI >25 (4, 3.3%), multiple pregnancies (6, 5.0%), older maternal age (14, 11.6%), or the use of oral hypoglycemic agents (26, 21.5%) as major risk factors. In terms of screening practices, 62 (51.2%) correctly identified the most comprehensive screening protocol for pregnant women with preexisting diabetes: before conception, in early pregnancy, and once per trimester thereafter. A notable proportion, 57 (47.1%), recognized that relying solely on a history of visual disturbances is not an adequate method for detecting DR, while 63 (52.1%) correctly disagreed that quick visual acuity and visual field assessments are the best screening tools. Instead, 74 (61.2%) acknowledged that detailed retinal examination by an ophthalmologist is the preferred screening approach. Finally, regarding treatment choices for PDR during pregnancy, 35 (28.9%) correctly recognized pan-retinal laser photocoagulation as a viable treatment option.

Table 3 reveals the participants’ attitudes toward DR. Nearly half of the participants, 58 (47.9%), strongly agreed that routine ophthalmic examinations before conception were a waste of resources, while 56 (46.3%) believed that such examinations during pregnancy were only necessary when vision was affected. Furthermore, 48 (39.7%) strongly agreed that ANC physicians have a limited role in preventing or treating DR. Additionally, only 32 (26.4%) strongly agreed that their institutions provide clear guidelines for managing DR in pregnancy.

**Table 3:** Attitude items ANC, antenatal care; DR, diabetic retinopathy

Item		Strongly agree	Agree	Neutral	Disagree	Strongly disagree
Routine ophthalmic examination for diabetic female patients before conceiving is a waste of resources.	N	58	39	13	4	7
	%	47.9	32.2	10.7	3.3	5.8
Routine ophthalmic examination for diabetic female patients before conceiving is time-consuming.	N	44	32	26	8	11
	%	36.4	26.4	21.5	6.6	9.1
Ophthalmic examination during pregnancy is required only when vision is affected.	N	56	34	15	12	4
	%	46.3	28.1	12.4	9.9	3.3
A pregnant lady with diabetes requires an ophthalmic examination by an ophthalmologist at each trimester.	N	20	25	30	18	28
	%	16.5	20.7	24.8	14.9	23.1
ANC physicians have not much of a role in the prevention and/or treatment of DR during pregnancy.	N	48	38	23	7	5
	%	39.7	31.4	19	5.8	4.1
The ophthalmic examination findings will not affect the patient’s ANC.	N	39	40	28	11	3
	%	32.2	33.1	23.1	9.1	2.5
There are clear instructions for dealing with DR in pregnancy provided by the institution where I work.	N	32	28	37	13	11
	%	26.4	23.1	30.6	10.7	9.1

Table 4 shows the assessment of participants’ practices. Fewer than one-third of the participants, 38 (31.4%), reported always educating patients about pregnancy as a risk factor for DR, while 47 (38.8%) stated they never assessed visual complaints during patient visits, which could hinder early detection. With regard to referral practices, only 39 (32.2%) consistently referred pregnant women with diabetes to ophthalmology, and the majority referred fewer than five women per month (102, 84.3%). Additionally, 49 participants (40.5%) consistently followed up on ophthalmic examination results, whereas 36 (29.8%) never did so. Moreover, 53 (43.8%) reported always following guidelines when managing DR, while 40 (33.1%) indicated they did not adhere to any specific guideline. Among those who followed guidelines, the most commonly used were those of the American College of Obstetricians and Gynecologists (61, 50.4%).

**Table 4:** Practice items ACOG: American College of Obstetricians and Gynecologists; ADA, American Diabetes Association; DR, diabetic retinopathy; SDCPG, Saudi Diabetes Clinical Practice Guidelines

Items	Responses	Frequency	Percentage
I educate my patients that pregnancy is considered one of the risk factors for developing DR.	Never	28	23.1
	Sometimes	55	45.5
	Always	38	31.4
I assess visual complaints at each visit for my pregnant lady who has diabetes.	Never	47	38.8
	Sometimes	44	36.4
	Always	30	24.8
I refer all pregnant ladies with diabetes to ophthalmology.	Never	37	30.6
	Sometimes	45	37.2
	Always	39	32.2
The number of pregnant women with diabetes that I refer for DR screening each month is approximately	Less than 5 women	102	84.3
	5-10 women	12	9.9
	More than 10 women	7	5.8
I follow up on the results of the ophthalmic examination after I refer my patient to ophthalmology.	Never	36	29.8
	Sometimes	36	29.8
	Always	49	40.5
I follow up on guidelines in approaching a pregnant lady regarding DR.	Never	38	31.4
	Sometimes	30	24.8
	Always	53	43.8
Specific:	ADA	12	9.9
	ACOG	61	50.4
	SDCPG	8	6.6
	I don’t follow any guidelines	40	33.1

**Figure 1:**
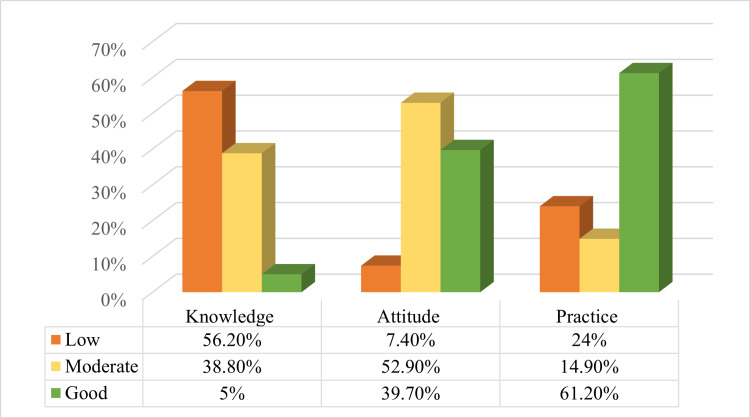
Scores of KAP KAP, knowledge, attitudes, and practices

Figure 1 shows that more than half of the participants, 68 (56.2%), demonstrated insufficient knowledge, while a substantial proportion, 47 (38.8%), exhibited moderate knowledge. Only a small percentage, 6 (5.0%), achieved good knowledge scores. In contrast, the attitude scores indicate a more positive outlook: 9 participants (7.4%) demonstrated low attitudes, 64 (52.9%) displayed moderate attitudes, and 48 (39.7%) demonstrated good attitudes. Regarding practice scores, 74 (61.2%) demonstrated good practice, 29 (24.0%) had insufficient practice scores, and 18 (14.9%) showed moderate practice.

Table 5 presents the analysis of sociodemographic characteristics in relation to KAP scores. No significant differences were observed between males and females in KAP scores. However, a statistically significant positive correlation was found between age and both attitude and practice scores, while no significant differences were noted in knowledge scores across age groups. Additionally, no significant differences in attitude or practice were observed based on nationality.

**Table 5:** Relationship between sociodemographic characteristics and KAP ^a^ Independent t test ^b^ ANOVA test ^*^ Significant ^#^ t value for the independent t-test and F value for the ANOVA test ANC, antenatal care; KAP, knowledge, attitudes, and practices

Sociodemographic characteristics		n	Knowledge				Attitudes				Practices			
			Mean	SD	Statistic value^#^	P-value	Mean	SD	Statistic value	P-value	Mean	SD	Statistic value	P-value
Sex^a^	Male	29	15.1	3.63	0.422	0.674	26.38	3.45	0.162	0.872	10.34	3.29	-0.113	0.911
	Female	92	14.72	4.49			26.23	4.64			10.42	3.3		
Age (years)^b^	Less than 30	32	25.25	3.76	0.695	0.262	12.53	3.45	0.834		8.69	3.22	1.43	0.002^*^
	30-50	82	26.55	4.63			15.79	4.24			10.96	3.03		
	More than 50	7	27.57	3.31			13.71	4.54			11.71	4.07		
Nationality^a^	Saudi	94	14.33	4.45	2.343	0.021^*^	26.27	4.34	0.007	0.994	10.14	3.44	-1.679	0.096
	Non-Saudi	27	16.48	3.19			26.26	4.56			11.33	2.51		
Specialty^b^	General practitioner	30	12.93	4.88	5.218	0.002^*^	25.3	4.94	2.117	0.102	9.23	3.4	2.779	0.044^*^
	Board resident	38	14.13	4.15			25.55	4.02			10.18	3.42		
	Specialist	30	16.73	3.74			27.73	4.41			10.9	3.01		
	Consultant	23	15.87	3.08			26.78	3.74			11.65	2.85		
Degree^b^	MBBS	45	12.98	4.47	5.4	0.002^*^	25.16	4.27	2.627	0.054	9.13	3.17	4.345	0.006^*^
	Diploma	8	16.25	4.77			29.38	3.81			11.75	4.2		
	Family medicine board-certified	59	15.61	3.75			26.71	4.27			10.93	3.02		
	Obstetrics and gynecology board-certified	9	17.44	3.17			26.11	4.83			12.11	2.98		
Years of experience in general^b^	Less than 1 year	42	14.19	3.92	7.383		26.29	4.09	0.209	0.811	10.19	3.37	2.583	0.08
	2-5 years	51	13.9	4.55			26.49	4.21			9.92	3.28		
	6-10 years	28	17.39	3.3			25.82	5.14			11.61	2.96		
Years of experience in ANC assessment^b^	Less than 1 year	45	13.87	3.81	6.621	0.002^*^	26.2	4.07	0.028	0.972	10.09	3.42	0.898	0.41
	2-5 years	47	14.23	4.58			26.38	4.99			10.28	3.3		
	6-10 years	29	17.21	3.66			26.17	3.86			11.1	3.04		
Working place^a^	Primary health care	16	14.56	6.54	-0.247	0.805	26.69	4.7	0.414	0.68	10.94	4.02	0.695	0.489
	Hospital	105	14.85	3.87			26.2	4.34			10.32	3.17		
Marital status^b^	Single	21	13.14	3.68	2.509	0.086	25.57	3.79	0.52	0.596	8.95	3.22	3.195	0.045^*^
	Married	97	15.24	3.96			26.36	4.53			10.77	3.19		
	Divorced/widowed	3	12.67	13.01			28	3			8.67	4.73		
Children^b^	Yes	93	26.63	4.45	3.269	00.042^*^	15.34	4.03	2.108	0.123	10.77	3.15	2.82	0.064
	No	7	23.43	4.2			12.71	7.36			9.86	4.41		
	NA	21	25.57	3.79			13.14	3.68			8.95	3.22		

Knowledge and practice scores increased with the level of professional qualification, with specialists and consultants demonstrating the highest scores. Similarly, knowledge scores were positively correlated with years of experience. Regarding marital status, single participants demonstrated significantly lower practice scores than married participants, while those with children had significantly higher knowledge scores compared to participants without children. The workplace setting (primary health care vs. hospital) did not significantly influence KAP scores.

## Discussion

In this study, the majority of participants were female, most of whom were between 30 and 50 years of age. A significant proportion were board residents or general practitioners with varying levels of training and expertise in DR management. Many held family medicine board certifications, while fewer specialized in obstetrics and gynecology. Experience in general practice and ANC was moderately distributed, with many participants reporting 2-5 years of experience. Most participants worked in hospital settings rather than primary healthcare centers, reflecting differences in patient exposure and resource availability.

Regarding knowledge, most ANC physicians in Al-Ahsa correctly recognized that pregnancy does not protect against DR but rather accelerates its progression, consistent with established medical literature [14,15]. Hormonal changes, including elevated human placental lactogen, estrogen, and progesterone, are well-documented contributors to DR worsening, as they increase vascular permeability and insulin resistance [16]. However, fewer physicians associated these hormonal fluctuations with direct vascular changes, highlighting a knowledge gap concerning the underlying pathophysiological mechanisms, which are well described in the literature [17]. With respect to diabetes types, some confusion was noted: only a small proportion correctly identified type 1 diabetes as carrying the highest risk for DR progression during pregnancy. This contrasts with evidence showing that pregestational diabetes, particularly type 1, significantly increases retinopathy risk due to longer disease duration and more severe metabolic dysregulation [18]. While most participants appropriately dismissed type 2 and gestational diabetes as the highest-risk categories, misconceptions persisted, possibly due to differences in clinical exposure or training.

Key risk factors for DR progression, including long disease duration, preexisting retinopathy, poor glycemic control, and hypertension, were generally well recognized by participants. However, misconceptions regarding BMI, maternal age, and oral hypoglycemic agents as primary risk factors suggest that some physicians overemphasize less critical variables, underscoring the need for clearer education on risk stratification. Screening practices showed variable adherence to evidence-based protocols. More than half of participants endorsed comprehensive screening (preconception and trimester-based), while a considerable proportion relied on suboptimal methods, such as patient-reported visual symptoms. This diverges from recommendations by the ADA and the International Federation of Gynecology and Obstetrics (FIGO), which emphasize routine ophthalmologic evaluation during pregnancy [19,20]. Encouragingly, many participants favored specialist-performed retinal examinations. However, recognition of pan-retinal laser photocoagulation as a treatment for PDR in pregnancy was low, despite strong evidence supporting its safety and effectiveness [21]. This likely reflects limited collaboration between obstetricians and ophthalmologists [22].

Overall, the study’s findings on KAP regarding DR in pregnancy highlight both strengths and areas for improvement, consistent with trends in Saudi Arabia and internationally. For example, Al-Rubeaan et al. reported that healthcare providers in the Kingdom often demonstrated suboptimal knowledge of diabetic complications, including DR, particularly in primary care where exposure to specialized management is limited [22]. Similarly, Alhejji et al. found that the mean overall knowledge score was only 2.6 ± 1.16 out of 4, with just 24.1% of participants referring diabetic patients according to the AAO guidelines, and only 36.9% educating patients on early detection of complications [5]. Thirunavukkarasu et al. also reported low KAP scores among physicians, with high scores observed in only 21.5%, 15%, and 29.2% of participants, respectively, aligning with the present findings [23].

Comparable international trends have been observed. In India, Singh et al. reported that only 32% of obstetricians and general practitioners had adequate knowledge about DR screening in pregnancy, attributing this to insufficient training and lack of standardized protocols [24]. In Nigeria, fewer than 50% of ANC providers were aware of the association between pregnancy and DR progression [25].

In contrast, participants in the present study demonstrated relatively favorable attitudes, with most reporting moderate to good attitudes toward DR screening and management. These findings are similar to those of Almotairy et al. in Riyadh, where physicians strongly agreed on the importance of screening but cited systemic barriers, such as time constraints and referral challenges, as obstacles to adherence [26]. A comparable gap between attitudes and practices was reported in a UK study by Scanlon et al., in which 78% of clinicians acknowledged the importance of DR screening during pregnancy, but only 45% followed guidelines due to workflow inefficiencies [27].

Practice scores in this study were comparatively higher, with over 60% of participants demonstrating satisfactory practices. This may reflect ongoing healthcare reforms in Saudi Arabia, such as Vision 2030 initiatives, which emphasize integrating diabetes and ANC services, including DR screening programs [28].

Strengths and limitations

This study provides novel insights into ANC physicians’ DR-related KAP in Al-Ahsa, Saudi Arabia, supported by a robust sample and detailed scoring. However, self-reported data may introduce reporting bias, and the focus on Al-Ahsa limits the generalizability of findings to other regions. Although the questionnaire was tailored to local contexts, it was not standardized to international benchmarks. Furthermore, clinical outcomes were not directly assessed.

## Conclusions

This study demonstrated gaps in knowledge regarding DR during pregnancy among ANC physicians in Al-Ahsa, Saudi Arabia, despite generally favorable attitudes and moderately adequate clinical practices. Greater awareness and adherence to DR screening protocols were observed among more experienced and specialized physicians. Although attitudes toward DR screening were largely positive, practical implementation remained suboptimal. A structured training program on DR in pregnancy, particularly targeting primary care and early-career physicians through continuing medical education, is strongly recommended. In addition, awareness campaigns, improved institutional protocols, and policy updates should mandate routine DR screening for all pregnant women with diabetes, in line with Saudi Arabia’s Vision 2030 healthcare goals.

## Appendices

Questionnaire

Appendix A

**Table 6:** Demographical data

Gender	Male
	Female
Age (in years)	
Nationality	Saudi
	Non-Saudi
Title	General practitioner
	Board resident
	Specialist
	Consultant
Degree	MMBS
	Diploma
	Family medicine board-certified
	Obstetrics and gynecology board-certified
	Obstetrics and gynecology fellowship
	Family medicine fellowship (women's health)
Years of experience in general	Less than 1 year
	2-5 years
	6-10 years
	More than 10 years
Years of experience in antenatal care assessment	Less than 1 year
	2-5 years
	6-10 years
	More than 10 years
Workplace	Primary health care centers
	Hospital
Marital status	Single
	Married
	Divorced/widowed
If married, do you have children?	Yes
	No
	NA

Appendix B

**Table 7:** Knowledge items * True answer VEGF, vascular endothelial growth factor This questionnaire was created by the authors.

Items	Yes	No	I don’t know
Pregnancy is considered protective against diabetic retinopathy.		*	
Pregnancy is a risk factor for diabetic retinopathy.		*	
Theories behind the progression of diabetic retinopathy in pregnancy			
Hormonal changes include increased plasma levels of human placental lactogen, oestrogen, and progesterone hormones.	*		
Vascular changes induced by the elevated levels of oestrogen, progesterone, and human placental lactogen.	*		
Is type 1 diabetes the most prone to developing diabetic retinopathy?	*		
Is type 2 diabetes the most prone to developing diabetic retinopathy?		*	
Is gestational diabetes the most prone to developing diabetic retinopathy?		*	
Which one contributes as a risk factor to the worse progression of diabetic retinopathy during pregnancy?			
Long duration of DM	*		
History of retinopathy before conception	*		
Poor glycemic control		*	
Hypertension, either preexisting or pregnancy-induced, and preeclampsia		*	
BMI more than 25		*	
Multiparity		*	
Older age		*	
Using an oral hypoglycemic agent		*	
Answer regarding the frequency of ophthalmic examination in diabetic pregnant ladies			
All pregnant ladies with preexisting diabetes should be screened for diabetic retinopathy once during pregnancy.		*	
All pregnant ladies with preexisting diabetes should be screened for diabetic retinopathy each trimester.		*	
All pregnant ladies with preexisting diabetes should be screened for diabetic retinopathy once before conceiving, in early pregnancy, and each trimester, then after accordingly.		*	
Is a history of visual disturbance the best method to identify diabetic retinopathy in pregnant women with diabetes?		*	
Quick visual acuity and visual field assessment is the best method to screen pregnant women with diabetes regarding diabetic retinopathy?		*	Detailed retinal examination
Is working with ophthalmologists the best method to screen pregnant women with diabetes regarding diabetic retinopathy?	*		
The treatment options for a pregnant lady with proliferative diabetic retinopathy during pregnancy?			
Observational		*	
Wearing glasses		*	
Pan retinal laser photocoagulation	*		
Focal laser		*	
Intravitreal steroids		*	
Intravitreal anti-VEGF		*	

*Appendix C* 

**Table 8:** Attitude items ANC, antenatal care This questionnaire was created by the authors.

Item	Strongly agree	agree	Neutral	Disagree	Strongly disagree
Routine ophthalmic examination for diabetic female patients before conceiving is a waste of resources.					
Routine ophthalmic examination for diabetic female patients before conceiving is time-consuming.					
Ophthalmic examination during pregnancy is required only when vision is affected.					
A pregnant lady with diabetes requires an ophthalmic examination by an ophthalmologist at each trimester.					
ANC physicians have no much role in the prevention and/or treatment of diabetic retinopathy during pregnancy.					
The ophthalmic examination findings will not affect the patient’s ANC.					
There are clear instructions in dealing with diabetic retinopathy in pregnancy provided by the institution where I work.					

Appendix D 

**Table 9:** Practice items ACOG: American College of Obstetricians and Gynecologists, ADA, American Diabetes Association; DR, diabetic retinopathy; SDCPG, Saudi Diabetes Clinical Practice Guidelines This questionnaire was created by the authors.

Items	Answers
I educate my patients that pregnancy is considered one of the risk factors for developing diabetic retinopathy.	Never
	Sometimes
	Always
I assess visual complaints at each visit for my pregnant lady who has diabetes.	Never
	Sometimes
	Always
I refer all pregnant ladies with diabetes to ophthalmology.	Never
	Sometimes
	Always
The number of pregnant women with diabetes that I refer for diabetic retinopathy screening each month is approximately	Less the 5 women
	5- 10 women
	More than 10 women
I follow up on the results of the ophthalmic examination after I refer my patient to ophthalmology.	Never
	Sometimes
	Always
I follow up guidelines in approaching a pregnant lady regarding diabetic retinopathy.	Never
	Sometimes
	Always
If (sometimes or always) specific:	ADA
	ACOG
	SDCPG
	I don't follow any guideline
